# Comparative proteomics analysis of degenerative eye lenses of nocturnal rice eel and catfish as compared to diurnal zebrafish

**Published:** 2013-03-20

**Authors:** Yi-Reng Lin, Hin-Kiu Mok, Yuan-Heng Wu, Shih-Shin Liang, Chang-Chun Hsiao, Chun-Hao Huang, Shyh-Horng Chiou

**Affiliations:** 1 Center for Research Resources and Development, Kaohsiung Medical University, Kaohsiung, Taiwan; 2 Institute of Marine Biology, National Sun Yat-Sen University, Kaohsiung, Taiwan; 3 Graduate Institute of Medicine, Kaohsiung Medical University, Kaohsiung, Taiwan; 4 Department of Biotechnology, College of Life Science, Kaohsiung Medical University, Taiwan; 5 Department of Biotechnology, Fooyin University, Kaohsiung, Taiwan; 6 Graduate Institute of Clinical Medical Sciences and Genomic and Proteomic Core Laboratory, Kaohsiung Chang Gung Memorial Hospital, Chang Gung University Kaohsiung, Taiwan; 7 Asia-Pacific Ocean Research Center, National Sun Yat- Sen University, Kaohsiung, Taiwan; 8 Institute of Biological Chemistry, Academia Sinica, Taipei, Taiwan

## Abstract

**Purpose:**

The aim of this study was to determine the lens crystallin diversity of degenerative eyes from the rice eel (*Monopterus albus*) and walking catfish (*Clarias batrachus*) as compared to that of zebrafish (*Danio rerio*) by using comparative proteomics methodologies. We endeavored to investigate the evolution of vertebrate lenses particularly concerning the functional loss of lenses in degenerative eyes of rice eels and catfishes living under an environment of perpetual darkness.

**Methods:**

Fish lenses were collected and homogenized to extract total soluble proteins. The protein mixtures were separated by one- and two-dimensional gel electrophoresis (1D or 2D gel), plus the newer gel-free shotgun proteomic strategy, followed by in-gel digestion and subjection of the digested protein bands or spots to liquid chromatography coupled with tandem mass spectrometry. The proteomics data were analyzed and compared based on the proteomics databank of zebrafish. The soluble lens protein solutions of three piscine species were also processed by gel-filtration chromatography and 1D sodium dodecyl sulfate–polyacrylamide gel electrophoresis for the comparison and validation of various crystallin families, e.g., α-, β-, and γ-crystallins.

**Results:**

In zebrafish eye lenses, γ-crystallin constituted about 71% and α- and β-crystallins comprised 30% of total lens proteins. In rice eel lenses, very little or almost no α-crystallins were detected and β- and γ-crystallins comprised more than 98% of total lens proteins. In catfish lenses, α- and β-crystallins comprised about 40% and γ-crystallin constitutes 60% of total lens proteins. It was of interest to find that α-crystallin was totally absent in the rice eel in contrast to the presence, albeit with very low amounts, of α-crystallin in similarly nocturnal catfish. The ratio of α-crystallin subunits (αA/αB) was found to be about 20:1 for the catfish lens, in great contrast to the ratio of about 3:1 found for most mammalian lenses. In contrast, β- and γ-crystallins were more abundant in lenses of these three piscine species, similar to mammalian lenses. By proteomics analysis, the most abundant β-crystallins were found to comprise a diverse group of βA1a, βA1–2, βA2a, βA2–2, βA4, βB1, βB2, and βB3 subunit crystallins; the monomeric γ-crystallin class contains γB, γD, γM2, γM3, γM5, γM7, γN–A, γN–B, γS1, and γS2 crystallins.

**Conclusions:**

In cave or nocturnal animals, the eye is sometimes reduced or eliminated because of adaptation to life in visual darkness. The comparative proteomics analysis of degenerative and normal lenses forms a firm molecular basis to investigate further the evolution of piscine lenses in the future. The total numbers of α-, β-, and γ-crystallins in the three fish species as revealed by the current proteomics methodology clearly indicate the complexity and diversity of crystallin species present in the piscine class of vertebrates. The unexpected finding that α-crystallin is absent in the degenerative eye lenses of rice eel may have some bearing on the chaperone function of α-crystallin in regard to its protective role of preventing protein aggregation in diurnal vertebrate lenses to maintain functional transparency.

## Introduction

The eye lens is a unique tissue designed for light transparency and image focusing in vertebrates. Eye lenses of vertebrates are composed of elongated fiber cells, of which approximately 90% of the total soluble proteins belong to three major classes of proteins, i.e., α-, β-, and γ-crystallins. Essentially, these crystallins can exist in the eye lens with little turnover throughout the entire lifespan, albeit with various degrees of posttranslational modifications on crystallin molecules [1,2].

Although living environment of fishes and most mammals are different, their eyes exhibit certain degree of similarity [1,3]. Recently, the zebrafish has been used as a suitable animal model [4,5] to study human eye diseases such as glaucoma [6], cataract [7], and retinal degeneration and regeneration [8,9]. Among these, cataract is the main focus for disease-related lens research. The transparency of eye lenses depends on the proper arrangement or association of lens proteins, i.e., various classes of α-, β-, and γ-crystallins inside lens fiber cells. The perturbation on the protein structure of lens crystallins by heat, chemicals, and other environmental stresses can lead to lens opacity due to spontaneous precipitation or crystallization of disturbed or denatured crystallin molecules [10].

In this study, we applied fast-evolving proteomics methodologies [11,12] to study some nocturnal fishes, including rice eel and walking catfish. Similar to blind cavefishes [13,14], these two teleosts, which possess degenerative eye lenses distinct from most common bony fishes [15,16], live in dim or totally dark environments such as muddy ponds, rice fields, and swamps. Many evolutionary viewpoints have been proposed to account for eye degeneration since Darwin’s treatise on *The Origin of Species* was published in 1859 [17]. The evolutionary mechanisms responsible for eye degeneration in cave-adapted animals have not been resolved. However, Strickler et al. [18] did suggest a possible correlation between downregulated αΑ-crystallin and eye lens degeneration in the cavefish. Two contrasting hypotheses invoking neural mutation or natural selection [14] have been advanced to explain eye regression, in spite of the fact that little or no experimental evidence has been presented in support of either theory.

Mainly attributable to the advent of emerging proteomics, the analysis and identification of complex protein mixtures in biologic tissues have recently become less tedious and more amendable to routine analysis [12,19]. In this study, we aim to characterize and compare the lenticular proteins from normal zebrafish and degenerative lenses of the rice eel and catfish by conventional one-dimensional (1D) and two-dimensional (2D) gel electrophoresis [20,21], together with newer gel-free shotgun proteomic strategy [19], followed by liquid chromatography coupled with tandem mass spectrometry (LC-MS/MS). Based on our results on the comparison and evaluation of crystallins in the lenses of these three species, we conclude that there exist some similarities and differences in crystallin expression patterns between normal zebrafish and degenerated rice eel or catfish lenses, one of the most prominent adaptive alterations being found in the quantitative differences of α-crystallin expression.

## Methods

### Materials

**Figure 1:**
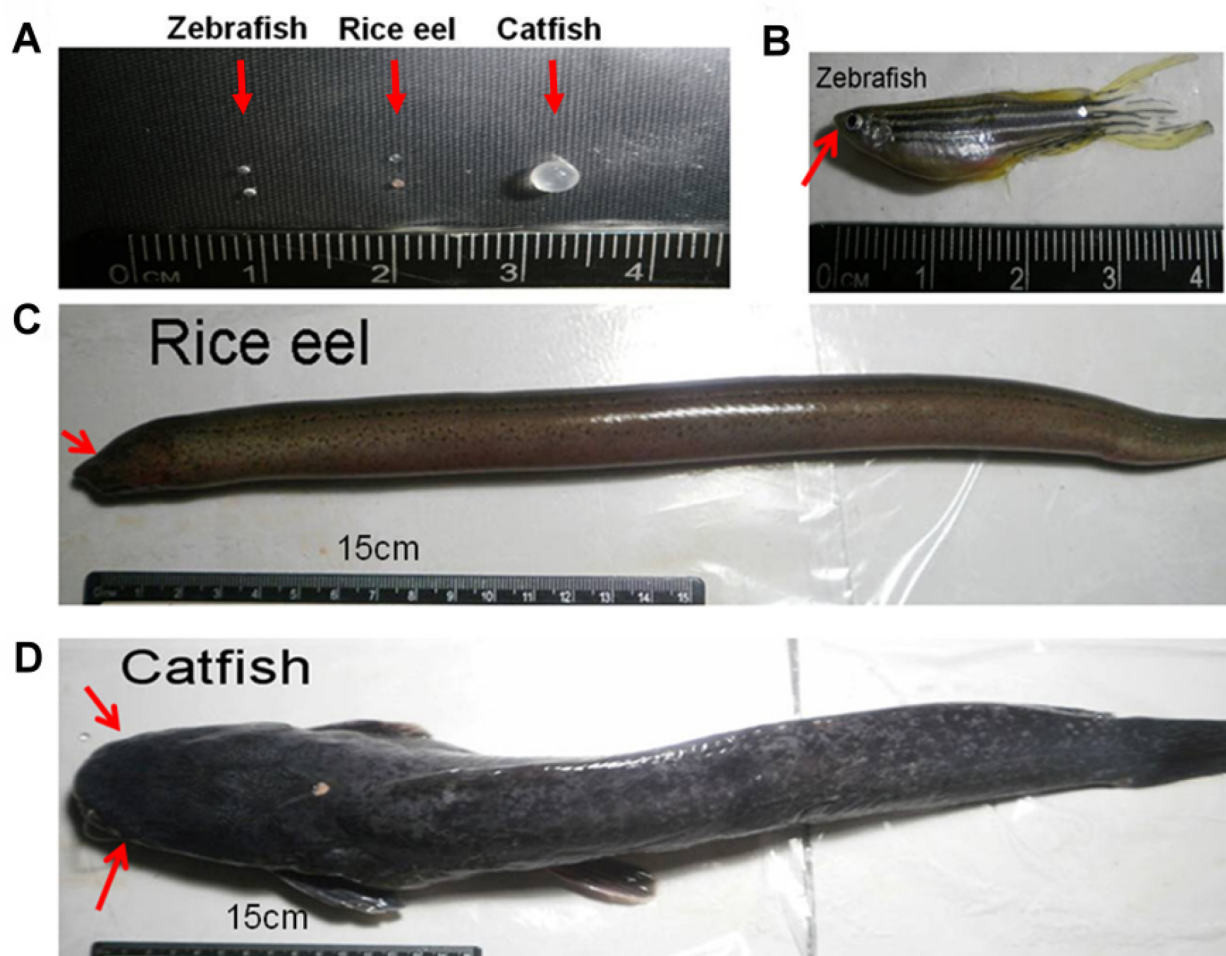
The lenses and body size of three piscine species were shown for comparison. **A**: The eye lenses isolated from zebrafish, rice eel, and catfish. The arrows indicate two small-size lenses for zebrafish and rice eel, and one big-size lens from catfish. The ruler with number marks shown at the bottom is used for size comparison. Body-size comparison among (**B**) zebrafish (**C**) rice eel and (**D**) catfish. The arrows indicate the location of eye lenses.

All zebrafish (*Danio rerio*) were about 6 months old and supplied from local aquarium stores. Rice eel (*Monopterus albus*) and walking catfish (*Clarias batrachus*) were also obtained from a local fish market in Kaohsiung, Taiwan. Rice eel, with the small eyes covered by a layer of skin (Figure 1), can burrow into the humid soil bottom for a period of days in the dry season without exposure to daylight.

### Extraction of soluble proteins from fish lenses

Eyeballs were kept and stored at −80 °C in a freezer before dissection. The lenses were removed from the adult fish eyeballs, homogenized, and suspended in the buffer of 20 mM Tris-HCl, pH 6.8 for the extraction of total lens. All animal protocols were approved by the Animal Care and Use Committee at Kaohsiung Medical University. Euthanasia of zebrafish, rice eel and catfish was carried out by treatment with tricaine (Sigma-Aldrich, St. Louis, MO) before protein extraction.

### One-dimensional and two-dimensional sodium dodecyl sulfate–polyacrylamide gel electrophoresis

The 1D sodium dodecyl sulfate–polyacrylamide gel electrophoresis (SDS–PAGE) was prepared in 5% and 15% (gel percentage) for stacking and resolving gels (Hoefer SEM 260 system, Amersham Pharmacia, Piscataway, NJ). Electrophoresis was run for 4.5 h at 80 V after loading about 20 μg protein in each sample lane. The gels were stained with Coomassie blue G-250. For 2D gel, the dried immobilized pH gradient (IPG) strips (Immobiline^TM^ Drystrip pH 3–10, 13 cm; GE Healthcare, Milwaukee, WI) were rehydrated with 250 μl reswelling solution (7 M urea, 4% NP-40, 2 M thiourea, 1 mg/l bromophenol blue) and 3 ml cover fluid (mineral oil) in a reswelling tray overnight (12–16 h). Lens extracts containing about 200 μg protein each in 0.5% IPG buffer and 1% DTT were then performed by isoelectric focusing (IEF) on IPG strips for the first-dimensional electrophoresis. IEF was performed initially at 300 V for 3 h, then 1,000 V for 3 h, and finally at 8,000 V for 6 h until 40 kV-h was reached at 20 °C on an electrophoresis apparatus (Ettan IPGphor 3). The IPGs of 1D gel analysis were then equilibrated for 20 min in 5 ml of equilibration solution I (0.01% bromophenol blue, 6 M urea, 2% SDS, 30% glycerol, 50 mM Tris-Cl [pH 8.8], and 30 mM DTT), followed by 20 min in 5 mL of equilibration solution II (0.01% bromophenol blue, 6 M urea, 2% SDS, 30% glycerol, 50 mM Tris-Cl [pH 8.8], and 135 mM iodoacetamide). Finally, IPG strips were placed on the top of 15% resolving gels sealed with 0.5% agarose for electrophoresis at the second-dimensional SDS–PAGE. The 2-D gel electrophoresis was carried out at a constant current of 22.5 mA for 6 h, and stained with Coomassie blue G-250.

### In-gel digestion

The Coomassie-blue stained 1D SDS–PAGE and 2D gels in triplicate for each sample were prepared for in-gel digestion as described previously [22]; the protein spots (2D gels) or bands (1D gels) were first excised and placed in 0.65 ml siliconized tubes. One hundred microliter buffers (25 mM ammonium bicarbonate [NH_4_HCO_3_]/50% acetonitrile [ACN]) were added to sample tubes and vortexed for 10 min. The supernatants of vortexed samples were discarded, and then dried with speed-vacuum centrifuge to complete dryness. Digestion was started by adding enzyme solutions containing 20 ng/μl of modified sequence-grade trypsin (Promega, Madison, WI) in 25 mM NH_4_HCO_3_ (pH 8) buffer to cover sliced and dried gel pieces, rehydrated on ice for 10 min, and then incubated at 37 °C overnight (about 16 h). The digested aqueous solutions were transferred into 0.65 mL siliconized tubes and extracted twice with 30 μl of 50% ACN/5% formic acid (FA). The extracted enzyme-digested samples were dried by centrifugation in a vacuum centrifuge for the removal of ACN and FA, redissolved in 10 μl of 5% ACN/0.1% FA and subjected to analysis by LC-MS/MS.

### Liquid chromatography coupled tandem mass spectrometry analysis from one-dimensional or two-dimensional gels

Electrospray MS was performed using (A) a Waters-Micromass electrospray ionization quadrupole-time of flight (Waters, Manchester, UK) [23,24], or (B) an HCT Ultra ETDIIIon-Trap Mass Spectrometer (Bruker Daltonics, Bremen, Germany) interfaced with DIONEX UltiMate^TM^ 3000 Nano and Cap system (Dionex, Sunnyvale, CA) capillary high-performance liquid chromatography system. A 100 × 0.075 mm C18 column (3.5 μm particle diameter) with mobile phases of A (0.1% FA in water) and B (0.1% FA in ACN) were used. The peptides were eluted at a flow rate of 0.4 μL/min with an ACN gradient, which consisted of 5%–10% B in 5 min, 10%–50% B in 25 min, and 50%–95% B in 4 min.

The spectra for the eluting fractions were acquired as successive sets of scan modes. The MS scan determines the intensity of the ions in the m/z range of 200 to 2,000, and a specific ion was selected for a tandem MS/MS scan. The former examined the charge number of the selected ion and the latter acquired the spectrum (Collision-Induced Dissociation [CID] spectrum or MS/MS spectrum) for the fragment ions derived by collision-induced dissociation. The centroid MS/MS data of enzyme-digested fragments from protein bands (1D gel) or spots (2D gel) were obtained by using MassLynx 4.0 software (Waters, Manchester, UK), and then submitted them to the bioinformatics search program MASCOT, which was set up to search through the NCBI databases based on zebrafish (*D. rerio*) with the following parameters: a mass tolerance of 0.2 Da for precursor and fragment ions; one missed cleavage allowed for trypsin digestion; carbamidomethyl cysteine as fixed modification; and oxidized methionine as optional modification. The resultant identification had a statistically significant (p≤0.05) peptide score (based on MS/MS spectra).

### Isolation of lens proteins by size-exclusion chromatography

The eye lenses of zebrafish, rice eel, and walking catfish were homogenized in 100 mM NH_4_HCO_3_ buffer (pH 7.4), containing 5 mM protease inhibitor (Complete Mini Protease Inhibitor Cocktail; Roche Molecular Biochemicals, Indianapolis, IN). The homogenized lens solutions were centrifuged at 27,000g for 1 h at 4 °C. The concentration of the supernatant was determined using the Bio-rad protein assay kit. One hundred microliters of supernatant containing about 200 μg protein was applied to high-performance liquid chromatography TSK-GEL G4000SW_XL_ (Tosoh Co., Tokyo, Japan) [25,26]. The crystallin fractions were eluted at a flow rate of 0.75 ml/min and monitored for absorbance at ultraviolet 280 nm. Molecular weight standards were used for calibration of molecular sizes of eluted proteins versus their respective elution volume for the chromatography. Eluted crystallin fractions were further analyzed by SDS–PAGE and protein bands identified by silver staining.

### Quantitation by ImageMaster analysis

2D gels were digitally imaged with Image Scanner (Amersham Pharmacia) and the quantities of protein spots on 2D gels were analyzed by ImageMaster^TM^ 2D Platinum Software (Version 5.0). 2D gels were analyzed for each fish lens sample and the relative abundance of selective protein spots was normalized to the total intensity of the entire gel. The proportion of each protein spot was calculated as the mean of determinations for three separate lens samples.

### Chaperone-like activity assay

Chaperone-like activities of total lens extracts from the three piscine species were analyzed by measuring the capability to prevent the aggregation of alcohol dehydrogenase denatured by heating treatment or the reduction of disulfide bonds in insulin, as described previously [27,28]. In brief, chaperone-like activity was studied based on the dithiothreitol-induced insulin reductive unfolding and chaperone-assisted refolding. The assay was carried out at 25 °C by recording the turbidity change of OD_360nm_ within 42 min upon the initiation of dithiothreitol-induced insulin aggregation or until the turbidity curve reaches a plateau.

### Shotgun strategy for gel-free proteomics analysis

The detailed protocol was modified from that of a previous report [19] and is included in Appendix 1.

## Results and Discussion

Although greater emphasis in biologic research is being directed toward a comprehensive global analysis of cellular systems, reliable and high-throughput proteomics analysis of proteins has not existed until the advent of current proteomics instrumentation. The study of proteins at the level of molecular and cellular systems by means of fast-evolving and state-of-the-art proteomics methodologies has provided a firm basis for understanding the complex proteome profiles of total protein mixtures from whole tissues or cells of various sources [29].

In this study, we have applied sensitive and high-throughput proteomic methodologies to study and compare the crystallin composition of three fish species in the piscine class of vertebrates. Fish represents the oldest and most diverse group of vertebrates [30]. The modern fishes comprise two major classes of piscine species, i.e., Osteichthyes or teleostean (bony) fishes, and Chondrichthyes or cartilaginous fishes (e.g., sharks and skates). In this study, we focus on the comparison of crystallin compositions between two nocturnal species, i.e., rice eel and catfish, and one diurnal species, zebrafish.

### Comparison of morphology and the lens sizes of the three piscine lenses

All of the three piscine species are freshwater fishes, with rice eel and catfish generally living in the muddy fields during daytime, and preying food at night. Regarding the body size, the zebrafish is smaller than the rice eel and catfish. For the lens sizes, the catfish is bigger than the other two (Figure 1). Although the rice eel is larger than the zebrafish in body size, its lens is much smaller than that of the zebrafish due to its degenerative eye lenses. Ratios of lens size versus body length for the three species we studied are about 0.1 cm/4.5 cm for zebrafish, 0.09 cm/50 cm for rice eel, and 0.35 cm/55 cm for catfish. The ratios appeared to follow the order of zebrafish > catfish > rice eel. It is to be noted that catfish, rice eel, and zebrafish of the teleostean class were found to possess lenses that are harder than those of evolutionarily higher classes of vertebrates such as mammals. Even though the increased hardness of lenses was found to be reflected by the respective decrease in water content inside human lenses [31], biochemical factors influencing the hardness of animal eye lenses are not clear and warrant further study. Catfish lenses were also found to be harder than those of rice eel and zebrafish, with the latter being the softest among the three species.

### Fractionation of lens proteins by size-exclusion chromatography

**Figure 2:**
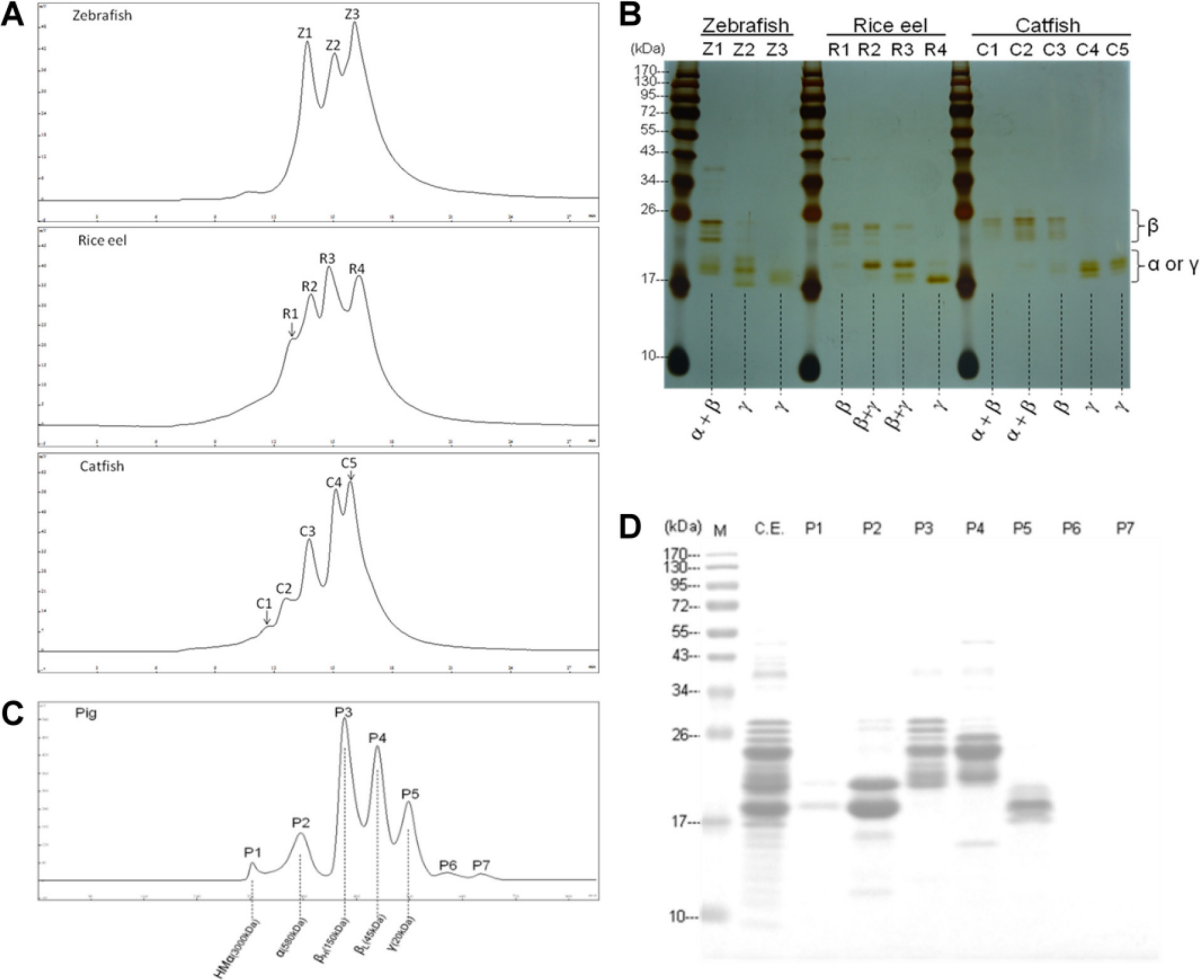
Gel-filtration chromatography and gel electrophoresis were used to fractionate lens extracts and characterize different crystallin families. **A**: Comparative gel-filtration chromatography on the TSK-G4000SW_XL_ size-exclusion column of lens extracts from the lenses of three piscine species (two nocturnal and one diurnal). Conditions were as described in “Materials and methods.” The column eluates (0.75 ml/tube per min) were monitored for absorbance at 280 nm. The 3–5 peaks (Z1-Z3, R1-R4, and C1-C5) in the middle of the figures correspond to the separated crystallin fractions of three fish lenses. The absorbances at 280 nm (ordinates) shown are relative concentrations in arbitrary units. **B**: Gel electrophoresis of the isolated crystallin fractions under denaturing conditions in the presence of 5 mM dithiothreitol (sodium dodecyl sulfate–polyacrylamide gel electrophoresis [SDS–PAGE]). Lanes Z1-Z3, R1-R4, and C1-C5 correspond to the three crystallin fractions of zebrafish, four crystallin fractions of rice eel, and five crystallin fractions of catfish in Figure 2A. The gels were stained with silver stain. The enclosed regions denoted by α, β, or γ on the right side indicate the subunit positions for α- and γ-crystallins with a molecular mass of about 20 kDa and β with molecular masses in a range of 24–32 kDa. The values in kDa for the far-left lane indicate the positions of protein markers with known molecular masses. **C**: Gel-filtration chromatography on the TSK-G4000SW_XL_ size-exclusion column of lens extracts from porcine lenses. The elution conditions are the same as those in Figure 2A. P1-P5 fractions correspond to the five mammalian crystallin fractions of HMα, α, βH, βL, and γ crystallins, respectively [32]. P6 and P7 are nonprotein small molecules. **D**: The isolated crystallin fractions in Figure 2C were analyzed by gel electrophoresis under denaturing conditions in the presence of 5 mM dithiothreitol (SDS–PAGE). P1-P5 correspond to five crystallin fractions and crude extract (CE) of porcine lenses shown in **C**. The gels were stained with Coomassie blue G-250.

**Figure 3:**
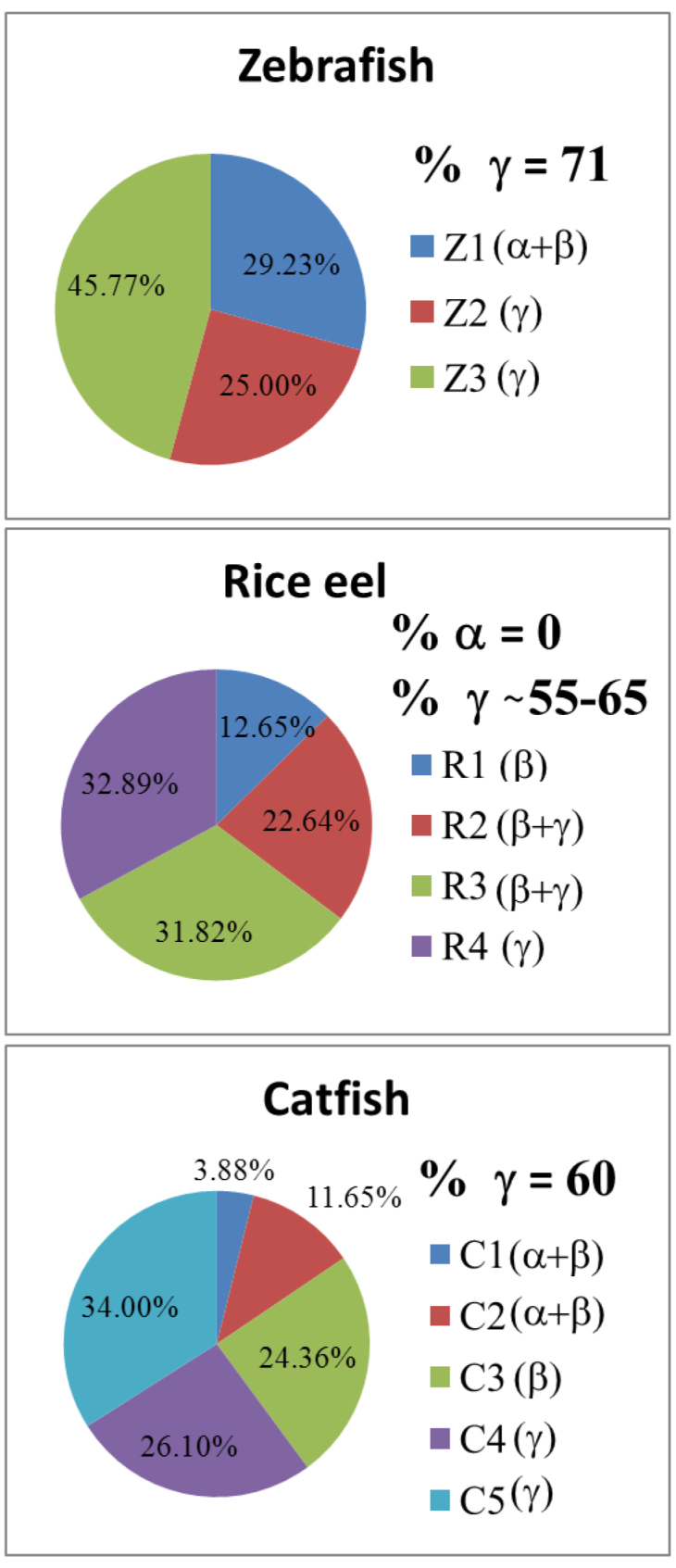
The percent abundance distribution of crystallin families in three piscine lenses was shown for comparison. γ-Crystallin was estimated to be 71%, ~55-65% and 60% for zebrafish, rice eel, and catfish, respectively, by peak areas calculated from Figure 2A. If we estimated percent abundance by densitometry of crystallin bands shown on sodium dodecyl sulfate–polyacrylamide gel electrophoresis (SDS–PAGE), α+β, and γ-crystallins were found to comprise about 29% and 71%, respectively, in the zebrafish eye lens. No α-crystallin was identified in rice eel, and β+γ crystallins comprised more than 98%. In catfish lenses, there is about 40% for α+β, and 60% γ-crystallins.

Figure 2A shows a typical elution pattern of lens extracts from three different species belonging to two nocturnal fish and one diurnal fish. Three well-defined peaks were obtained for the zebrafish lens, in contrast to four for the rice eel and five for the catfish. It is of interest that, in contrast to higher vertebrates, the first peak of the zebrafish elution pattern contains α-crystallin, as well as the aggregated form of β-crystallin (Figure 2B); this is also the case for the fractionation of catfish lenses, but is not common for higher classes of vertebrates, including reptiles, birds, and mammals [25,26,32,33]. It is also of interest that in contrast to zebrafish, catfish and rice eel contain very little α-crystallin, as revealed by the absence of α-crystallin subunit bands of about 20 kDa in SDS–PAGE (Figure 2B). In Figure 2C,D we show a comparative separation of porcine lens proteins into five well-defined fractions of HMα, α, βH, βL and γ crystallin classes similar to our previous characterization of bovine lens extract [32]. The proportions for each piscine crystallin fraction (Figure 3) are calculated based on the area of chromatographic peaks of each crystallin class as compared with the well-defined fractions of HMα (6.6%), α (31.4%), βH (24.6%), βL (23.0%), and γ (14.3%) for mammalian lenses. One salient feature of the crystallin contents in bony fishes of the piscine class is that the γ-crystallin contents of fishes are much higher than those of mammalian species such as the porcine γ-crystallin reported in this study (Table 1 and Figure 3). Our superior and reproducible resolution of the crystallins allowed us to make a systematic comparison between the crystallins of different classes. The well-defined and characteristic distribution of subunit compositions for each isolated fraction (Figure 2A-D) justified the use of size-exclusion gel in the general characterization and classification of lens proteins from various vertebrate and invertebrate species [33].

**Table 1:** Percent abundance of each crystallin fraction in three piscine species.

**Species**	**Molecular mass (kDa)**	**% abundance**
**Zebrafish**		
Z1	88.9	29.2%
Z2	34.5	25.0%
Z3	17.4	45.8%
**Rice eel**		
R1	141.8	12.7%
R2	77.2	22.6%
R3	41.7	31.8%
R4	15.0	32.9%
**Catfish**		
C1	331.5	3.9%
C2	181.2	11.7%
C3	82.6	24.4%
C4	33.0	26.1%
C5	20.1	34.0%

Molecular masses of fractions separated by size-exclusion chromatography were determined based on calibration by using protein markers. Percent abundance was estimated by the peak area of each fraction.

### Gel-based proteomic analyses of piscine lens extracts

**Figure 4:**
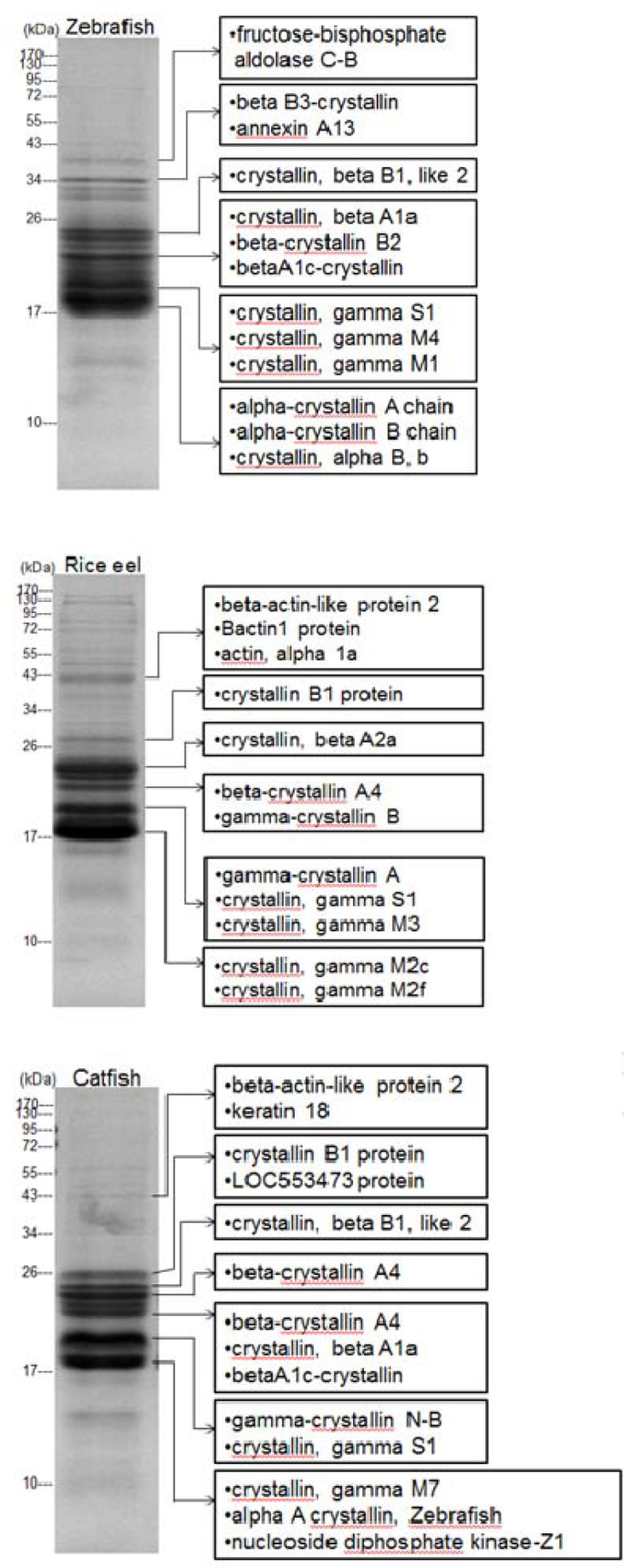
Comparative proteomics analysis identified lens proteins from zebrafish, rice eel, and catfish by sodium dodecyl sulfate–polyacrylamide gel electrophoresis (SDS–PAGE) followed by nano-liquid chromatography coupled tandem mass spectrometry (nanoLC-MS/MS). In the right panel, protein and peptide bands identified in the zebrafish databank with different expression levels are indicated by arrows. In comparison with zebrafish lens proteome, α-crystallin proteins in the rice eel and catfish lens were found to significantly decrease in expression levels as compared to zebrafish lens.

**Figure 5:**
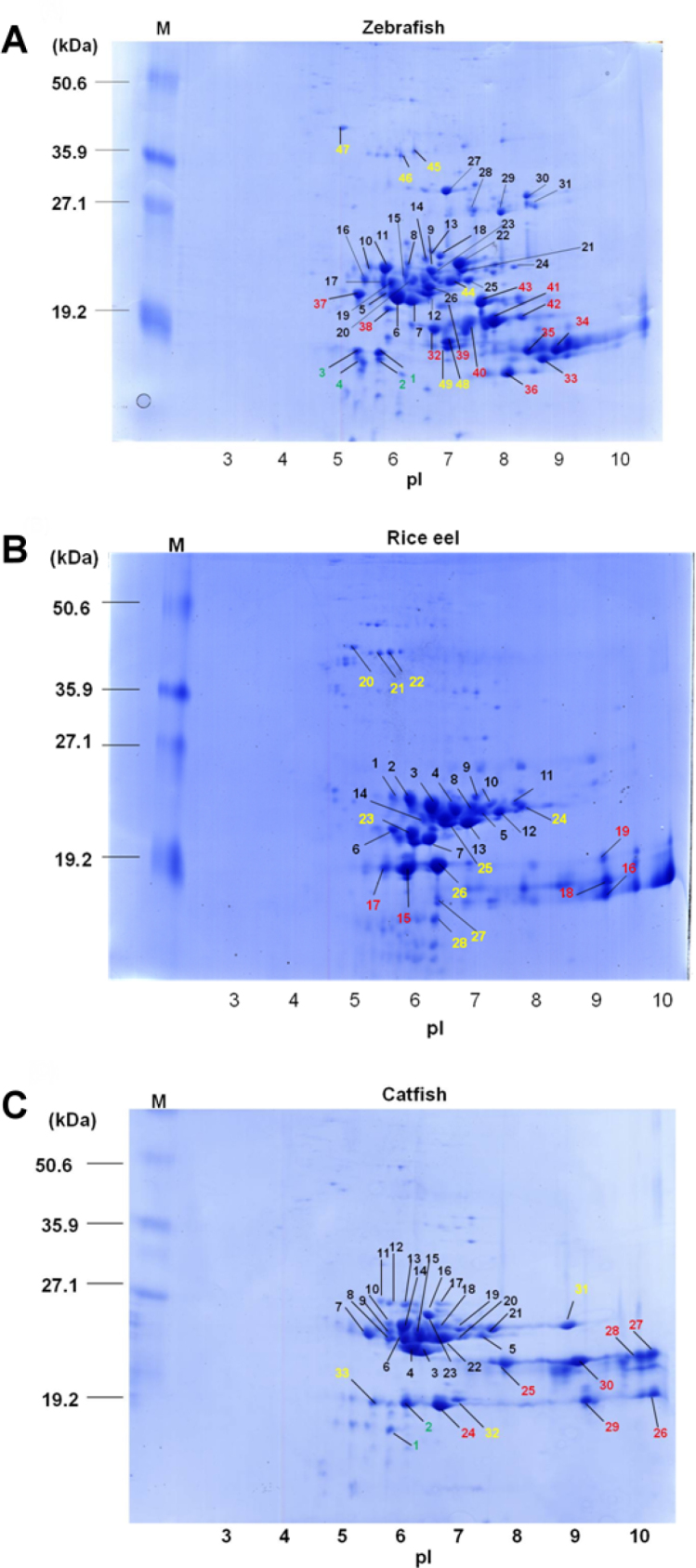
Two-dimensional gel patterns of piscine lens proteins. (**A**) Zebrafish, (**B**) rice eel, and (**C**) catfish. Total protein (200 μg) in each sample was loaded onto immobilized pH gradient (IPG) gel strips (pH 3–10 Nonlinear, 13 cm). The procedures were as described previously [34]. After electrophoresis, the gels were fixed in 40% methanol and 10% acetic acid and stained by Coomassie blue G-250. The IPG strips were rehydrated, and after isoelectric focusing (IEF), subjected to two-dimensional (2D) electrophoresis. Protein spots marked with numbers were further identified by nano LC-MS/MS and listed in Table 2. The result is representative of three independent 2D experiments for each fish species. Identified crystallins by proteomic analysis were denoted by green (α-crystallin), black (β-crystallin), red (γ-crystallin), and yellow (noncrystallin proteins).

The global protein-expression profiles of piscine lenses were analyzed using 1D (Figure 4) or 2D (Figure 5) gel electrophoresis. In this study, we first performed proteomics analysis on conventional 2D gel. The isoelectric point ( p*I*) range for the first-dimension isoelectric focusing (IEF) in 2D gel was 3–10, and the second dimension SDS–PAGE was run at 15% polyacrylamide gel (Figure 5). The proteomics analysis showed that more than half of lens proteins located on the weakly basic and low molecular weight regions (6.5 < p*I* <7.5 and 18,000 < molecular weight <32,000), corresponding mostly to β- and γ- crystallins. The proteomic analyses for zebrafish, rice eel, and catfish showed that we had positively identified about 49, 28, and 33 protein spots (No. 1–49, No. 1–28, No. 1–33 for lens proteins of zebrafish, rice eel, and catfish, respectively), as confirmed and verified by LC-MS/MS (Table 2).

**Table 2:** Identified members of three major crystallin classes in piscine eye lenses by 2D gel proteomic strategy

**Identified crystallins**	**Zebrafish**		**Rice eel**		**Catfish**	
	spot #	Intensity (%)	spot #	Intensity (%)	spot #	Intensity (%)
α A	3(1-3)	7.95	n.d.		2(1,2)	6.21
α B	1(4)	2.12	n.d.		n.d.	
β A1a	3(5- 7)	7.80	n.d.		2(3,4)	7.66
β A1-2	n.d.		n.d.		1(5)	2.88
β A2a	2(8,9)	4.36	5(1-5)	19.51	n.d.	
β A2-2	2(10,11)	4.37	n.d.		n.d.	
β A4	1(12)	2.67	2(6,7)	10.55	n.d.	
crystallin B1	5(13-17)	8.56	4(8-11)	10.86	13(6-18)	35.74
β B1	4(18- 21)	8.34	3(12-14)	10.14	3(19-21)	8.64
β B2	5(22-26)	8.67	n.d.		2(22.23)	6.71
β B3	5(27-31)	8.91	n.d.		n.d.	
γ B	1(32)	1.88	1(15)	5.17	n.d.	
γ D	n.d.	.	n.d.		1(24)	4.22
γ M2(predicted)	n.d.		n.d.		4(25-28)	11.92
γ M2c	1(33)	1.65	n.d.		n.d.	
γ M3	2(34,35)	4.44	1(16)	3.65	1(29)	3.08
γ M5	n.d.		3(17-19)	9.34	n.d.	
γ M7	1(36)	2.59	n.d.		n.d.	
γ N-A	1(37)	2.47	n.d.		n.d.	
γ N-B	1(38)	2.30	n.d.		n.d.	
γ S1	4(39- 42)	8.84	n.d.		1(30)	3.57
γ S2	1(43)	2.96	n.d.		n.d.	
others(unknown)	6(44-49)	9.12	9(20-28)	30.77	3	9.38
total	49	100	28	100	33	100

Intensity of protein spots was determined by *ImageMaster* analysis, followed by LC-MS/MS analysis on excised protein spots on 2D gels.

Four protein spots in the 2D gel of zebrafish were identified as αA-crystallin (protein spots #1–3) and αB-crystallin (protein spot No. 4), respectively. We also detected 27 protein spots that belong to the class of β-crystallins (protein spots No. 5–31), and 12 protein spots that belong to the class of γ-crystallins (protein spots No. 32–43). It is noteworthy that no protein spots corresponding to α-crystallins for rice eel were detected. We found 14 protein spots that belong to the class of β-crystallins (protein spots No. 1–14), and 5 protein spots in the class of γ-crystallins (protein spots No. 15–19). On the other hand, two protein spots located on the acidic region on the 2D gel of nocturnal catfish were identified as αA-crystallin (protein spots No. 1–2). We also detected 21 protein spots that belong to the class of β-crystallins (protein spots No. 3–23), and 7 protein spots to the class of γ-crystallins (protein spots No. 24–30). In Table 2, we have also listed 6, 9, and 3 protein spots (denoted as *others*) as anomalous proteins shown in 2D gels of zebrafish, rice eel, and catfish, respectively, because their identities cannot be revealed through searching and comparison with those identified crystallins deposited in the database of zebrafish.

### Comparison of gel-based one-dimensional or two-dimensional gel proteomics

In our proteomic study of porcine lens proteins [34], we encountered poor solubility of some proteins in pre-LC-MS/MS 2D gel separation. To improve the detection sensitivity for low-abundance proteins, fractionation of lens proteins was thus performed directly on the total extracts of three piscine lenses by 1D SDS–PAGE gels (Figure 4) instead of prerunning first-dimensional IEF in 2D gel without adding the strong protein denaturing and solubilizing agent of SDS (Figure 5). The proteins were separated into more than 10 different protein bands or zones from the total protein mixture each of three piscine lenses (Figure 4). In comparison with zebrafish lens-proteome, α-crystallin was found to be almost absent in the rice eel lens, similar to that observed in the 2D gel analysis described above. Loss of specific crystallins analogous to the missing α-crystallin from degenerative eyes for rice eel was also reported for lenses of some species under specific or pathological conditions, such as mutation-induced congenital cataract formation [35-37]. This pointed to the fact that differential crystallin expression under environmental stresses or pathological conditions may result in degenerative or deteriorating crystallin formation in the lenses of nocturnal rice eels.

It should be noted that 1D gel is methodologically less tedious and more time saving than 2D gels, being able to afford a respectable and extensive protein separation suitable for protein identification analysis after LC-MS/MS (Figure 4**)**. The unambiguous identification of some major crystallin species of β- and γ-crystallin classes were confirmed and verified in addition to α-crystallins reported previously.

Although 2D gel electrophoresis coupled with tandem MS has been considered as the method of choice in conventional proteomics study [38], only up to about 2,000 individual polypeptide chains at most can be resolved on a single 2D gel [39,40]. The number of detected proteins is still relatively small as compared to the whole proteome corresponding to the human genome, which encodes about 20,000-30,000 proteins. The 2D gel analysis is especially under-representative of some special classes of proteins, such as low-abundance transcription factors and membrane proteins [40-42], because of the low solubility of these classes of proteins in the first dimensional IEF protein separation of 2D gel electrophoresis in the absence of protein-solubilizing SDS detergent.

### Comparison of major lens-crystallin families of the three piscine species

The lens crystallins of vertebrates form a complex group of highly conserved structural proteins with distant evolutionary relationships [43]. To date, most physicochemical studies on the characterization of crystallins have placed more emphasis on species of higher vertebrates, with relatively fewer reports on lenses from the lower aquatic vertebrates, i.e., various classes of fishes [44]. The piscine lenses are usually spherical and hard as compared to the more flexible and soft lenses found in the avian class. The hardness of lenses has been shown to be related to the state and content of water, i.e., degree of hydration, in different lenses of vertebrate species [45]. The poor solubility and susceptibility of lens proteins from most fishes to denaturation have hampered detailed biochemical characterization of piscine crystallins under nondenaturing conditions [46,47].

In this report, we have adopted a systematic and general approach to isolate and characterize piscine crystallins of three different teleostean species by the proteomics approach to shed some light on the development and evolution of distinct crystallin families in piscine lenses. Herein, we focus on the analysis of the three prominent crystallin families, i.e., α-, β-, and γ-crystallins.

### Quantitative analysis of α-crystallin

α-Crystallin constitutes a major class of lens proteins present in all vertebrate eye lenses [48,49]. Native α-crystallin from mammalian lenses is commonly isolated as a large water-soluble aggregate with a molecular mass of about 600–800 kDa. It consists of two homologous subunits, αA and αB, of about 55%–60% sequence identity, each with a molecular mass of 20 kDa and in a binding ratio (αA/αB) of about 3:1 for most mammalian lenses [50]. Recently, α-crystallin—and especially its αB subunit—has received a lot of interest and attention because it was shown to possess structural and functional similarities to small heat-shock proteins [51,52]. Moreover, in vitro studies of α-crystallin indicated a chaperone-like activity associated with this lens protein [53,54]. Fewer studies on α-crystallin of piscine lenses have been reported due to its relative lower abundance as compared with β and γ crystallins in fish lenses. Therefore, it is deemed important to reevaluate the proportion of each major crystallin family in fish lenses of these three teleostean species (Figure 3). As described above, we found three protein spots of αA and one spot of αB for zebrafish; two spots of αA for catfish; and the absence of αA and αB for rice eel (Table 2 and Figure 4, Figure 5). Protein spot #4 in 2D gel of zebrafish lens was identified through search in NCBI database and listed as α B chain without specifying its being α Ba or α Bb. Quantitation of protein spot #4 in 2D gel (Table 2) of zebrafish lens is therefore either αBa or αBb. We do not know why we detect only one αB chain. However, we did detect two αB chains by 1D gel and shotgun proteomics (see Tables in Supplemental Materials on 1D gel and shotgun proteomics).

It is to be noted that the presence of more than one spot per crystallin member of the α-, β-, or γ-crystallin family in 2-D gel usually reflects truncated crystallin fragments or posttranslational modifications. However, a recent genomic study [55] indicated the existence of two αB-crystallins in the zebrafish, one lens specific (αBa-crystallin) and one ubiquitous (αBb crystallin). Determining the function of αA- or αB-crystallin in the piscine [33] and amphibian [56] lenses has been difficult because pure αA- or αB-crystallin is difficult to obtain from the above-mentioned size-exclusion chromatography. α-Crystallin and aggregated β-crystallins are always eluted together in the void volume of high-molecular-weight fraction. This also makes the accurate estimation of percent abundance of α-crystallin in fish lenses more difficult than that for γ-crystallin (Figure 3). In the literature, the reported percent abundance for piscine lenses also varies. For instance, the percent abundances of α-crystallin estimated previously by conventional protein analysis [57] were higher than that (7.8%) of a recent report employing a proteomic approach coupled with the densitometry of Coomassie stained 2D gels [58].

In our previous characterization of catfish α-crystallin from the same walking catfish (*C. batrachus*) as the species used in this study [27], it was found that in contrast to mammalian lenses with a subunit association ratio (αA-crystallin/αB-crystallin) of about 3:1, α-crystallin from catfish lens showed a ratio of about 19:1. This could also account for the difficulty of detecting catfish αB-crystallin in the current proteomics analysis. On the other hand, the possibility that the concentration of α-crystallin is too low or posttranslationally modified in the degenerated eye lenses of rice eel cannot be excluded. We have therefore investigated the chaperone-like activity of α-crystallin based on total lens extracts of three fish lenses. It is as expected that the chaperone activity of zebrafish is higher than that of catfish, which in turn is also much higher than that of rice eel, which exhibits no detectable activity (unpublished results).

It has been reported that αA-crystallin of zebrafish is a chaperone protein that can keeps the γ-crystallin of mutant zebrafish lenses soluble [7]. In contrast, the evolutionary and functional basis for the lower abundance of αB crystallin and the apparent absence of αA and αB crystallins in the degenerative lenses of catfish and rice eel, respectively, are especially intriguing; this topic remains elusive and deserves a further detailed study in the future employing genomics approaches.

### Quantitative analysis of β- and γ-crystallins

By proteomic analysis, the most abundant β- and γ-crystallins were found to comprise a diverse group of heterogeneous classes of βA1a, βA1–2, βA2a, βA2–2, βA4, βB1, βB2, and βB3 subunit crystallins, and γB, γD, γM2, γM3, γM5, γM7, γN-A, γN-B, γS1, and γS2 monomeric crystallins of the γ-crystallin class (Table 2). As we reported previously using a conventional protein chemistry methodology [33] and gene cloning [59], the class of carp γ-crystallin with high methionine content (γM-crystallin) was also found in most teleostean fishes, similar to those found for the diverse γ- and γS-crystallins of catfish [60,61] and γMs-crystallins of zebrafish [58], which is the most abundant crystallin family and comprises more than 30% of the lens.

In zebrafish, five βA-crystallins and three βB-crystallins comprised about 36% of total proteins in the zebrafish lens [58]. Many of the β-crystallins were extensively modified; for example, βB1 and βB2 were identified as 11 and 8 spots, respectively. Similar to the α-crystallin family, modified β-crystallins with different masses are probably due to truncations and other modifications with similar mass and the different isoelectric points may be due to phosphorylation or other modifications. In this pilot study, we did not pursue protein modifications of these nocturnal lenses due to scarcity of the lens samples for the top-down proteomic strategy [12]. There is always an issue for proteomics analysis of novel tissues lacking the complete genomic or proteomic databases such as rice eel and catfish regarding the possible false-negative results and discrepancies of protein components reported by different groups [58]. Therefore, for βB3 crystallin, which was found to be missing or reduced in rice eel or catfish, this may be due to the false negative identification (for rice eel) or reduced expression of existing crystallins with functional degeneration in adaptive dark environments (for catfish). However, we are confident that the positive identification of γ-B and γ-D is real and not artificially false identification, because they were revealed through widely accepted standard *Mascot* software analysis with high scores (Appendix 1). The analytical methodology for the estimation of the accuracy in peptides and their corresponding protein identifications made by MS/MS and database search has always been a big issue in proteomics analysis and identification. Other factors that may contribute to the ambiguity and uncertainty in protein identification could arise from the pre-MS/MS methodologies or protocols for sample treatment before protein separation, such as 1D gel, 2D gel, or shotgun proteomics strategies. Current state-of-the-art shotgun proteomics techniques may allow the sensitive identification of parent proteins from individual peptides in many complex protein mixtures of cell or tissue extracts. Specifically, shotgun proteomics is more sensitive than 2D gel electrophoresis for the separation and detection of proteins with low abundance.

The β- and γ-crystallins are evolutionarily related families of proteins that make up a large part of the refractive structure of the vertebrate eye lens [62]. Each family has a distinctive gene structure that reflects a history of successive gene duplications. Recently, Wistow et al. [63] made a survey of γ-crystallins expressed in mammal, reptile, bird, and fish species, which has resulted in the important discovery of γN-crystallin, an evolutionary bridge between the β and γ families. In all species examined, γN-crystallins have a hybrid gene structure, half β and half γ, leading to the supposition that they may be the “missing link” between β and γ crystallin lineages.

The γ-crystallin, unlike other lens crystallin, is monomeric in solution, and has the highest sulfhydryl content of all crystallins. X-ray crystallographic investigation by Summers et al. [64] has shown that the protein has a symmetric structure of two globular domains packed together with a single connection. It is suggested that the stability of γ-crystallin may be due to the interaction of polarizable amino acid groups and sulfur-containing residues such as methionine present in the core of each domain. Prediction of secondary and tertiary structure of carp high-methionine γM-crystallin by computer graphic simulation [65] has shown that carp γ-M1 crystallin comprises 22 methionine residues (12.4%), with 6 buried inside and 16 exposed on the surface. Carp γ-M2 crystallin with 24 methionine residues (14%) showed an essentially similar distribution pattern of methionine residues on the protein surface. Interestingly, most hydrophobic methionine residues are located on the protein surface with only a few buried inside the protein surface or in the interface between two motifs of each domain. The exposed hydrophobic and polarizable methionine cluster on the protein surface may have a bearing on the crystallin stability and dense packing in the piscine species, and probably also provides a malleable nonpolar surface for the interaction with other crystallin components for the maintenance of a clear and transparent lens.

There has been speculation [66] about the role of methionine in the regulation of the surface polarity of proteins as judged by the unique properties of the thioether sulfur atom in this generally hydrophobic amino acid. Incorporation of multiple methionine residues into carp γ-crystallins may therefore represent one efficient way of optimizing various crystallin interactions and recognitions in the intact lens. The existence of unusually high levels of methionine residues on the crystallin surface suggests that these hydrophobic amino acids with polarizable sulfur atoms might play a significant role in the recognition and interaction of crystallin molecules in the piscine lenses. Previous studies concerning the cold-adapted Antarctic toothfish [67,68] also described several methionine-rich γM-crystallin isoforms, of which the high methionine residues may have predisposed the toothfish lens to biochemically attenuate γ-crystallin hydrophobicity, thereby allowing for cold adaptation. Reduced structural constraints upon γM crystallins due to the presence of high methionine residues could have allowed for greater evolutionary plasticity, resulting in increased polydispersity of γ-crystallins contributing to the cold stability of the Antarctic toothfish lens. The complete lens proteome of zebrafish has indeed provided good model system to study investigations of vertebrate lens development, function, and diseases [58]. The similarity and differences of lens crystallins present in the three teleostean species studied herein will certainly provide some insights into the molecular basis for the structural and functional evolution of different crystallin families in fishes and other vertebrates. Future studies will be needed to further elucidate the posttranslational modification of these normal and degenerative piscine lens systems. The preliminary proteome map provided here lays a firm foundation for these investigations.

### Conclusions

The eye lens is an extraordinary tissue in terms of its development and evolution. In cave or nocturnal animals [13,14], the eye is sometimes reduced or eliminated as a consequence of adaptation to life in visual darkness. How the cavefish and rice eel adapted to develop degenerative eye lenses under dim or totally dark environments has remained an interesting evolutionary issue since Darwin’s time. In *The Origin of Species*, Darwin found no reason to invoke natural selection to explain the loss of eyes in cave animals [17]. As a result, the evolutionary mechanisms responsible for eye degeneration in cave-adapted animals have remained elusive. Opposing hypotheses invoking neural mutation [69] or natural selection [70], each with certain genetic and developmental expectations, have been advanced to explain eye regression, although little or no experimental evidence has been provided to support or refute either hypothesis. In this study, we have performed a comparative proteomics analysis on eye lenses of nocturnal rice eel and catfish as compared to diurnal zebrafish. The results of this analysis form a molecular basis to investigate further the evolution of piscine lenses in the future. The total α-, β-, and γ-crystallins in the three fish species analyzed by current proteomics methodology clearly indicate the complexity and diversity of crystallin species present in the piscine class of vertebrates. It is noteworthy that the unexpected finding that α-crystallin is absent in the degenerative eye lenses of rice eel points to the fact that α-crystallin acting as a chaperone protein may be essential in diurnal vertebrate species to protect lens proteins from aggregation and to maintain functional transparency of the lens under varied environmental conditions. A detailed study on the correlation of the chaperone function and eye degeneration in rice eel certainly warrants future investigation.

## Appendix 1. Supplemental Methods and Results.

To access the data, click or select the words “Appendix 1.”
